# Exploring the usability of simulated patient methodology in dental clinics in Western Australia: A pilot survey

**DOI:** 10.1002/cre2.906

**Published:** 2024-07-05

**Authors:** Viduni Liyange, Xin Rong Low, Joon Soo Park, Hien C. Ngo, Rhonda Clifford, Liza Seubert

**Affiliations:** ^1^ UWA Dental School The University of Western Australia Nedlands Western Australia Australia; ^2^ International Research Collaborative—Oral Health and Equity The University of Western Australia Crawley Western Australia Australia; ^3^ School of Allied Health The University of Western Australia Crawley Western Australia Australia

**Keywords:** dental practices, dental staff, simulated patient methodology, Western Australia

## Abstract

**Objectives:**

This study aimed to explore the dental staff knowledge of simulated patient methodology and support for its use to investigate dental staffs' triaging ability.

**Material and Methods:**

Staff at dental practices in Western Australia were invited to participate in a cross‐sectional online questionnaire, consisting of demographic questions, questions on triaging, and knowledge of simulated patient methodology. Descriptive and parametric tests were undertaken for quantitative data; qualitative responses were thematically analyzed.

**Results:**

Of the 100 participants, most were female (71%), aged 25−39 years (57%), dentists (46%), and worked in private practices (60%). While 82% of participants triaged dental appointment enquiries, only 26% had heard of simulated patient studies. The majority (66%) of participants spent 1−5 min when triaging appointments and less than half (29%) asked about medical history, aggravating or alleviating factors. Although there was a general positive attitude toward use of simulated patient methodology to investigate practice, some concerns were identified.

**Conclusions:**

The findings of our exploratory study suggests that there may be a potential for utilizing simulated patient studies to improve the care of patients by dental receptionists in general dental practices.

## 1 INTRODUCTION

A systematic triage approach should be unbiased and consistent; it should also ensure that all patients have equal access opportunities to dental care (Heggie, 2019). The receptionists at a dental practice are the first point of contact for patients, and they play an important role in ensuring that patients are able to access care in a timely manner (Kirton et al., 2020). Although they may be the first port of call for patients in a dental practice, dental reception is an unregulated profession and does not require specific training. Dental reception staff appear to have a lack of clinical knowledge, and, as a result, there are many pitfalls associated with dental receptionists triaging patients (Kirton et al., 2020). There are currently no simulated patient studies that have evaluated receptionist triaging abilities in private, general dental practices. In Western Australia, private dental practices are accredited based on whether they adhere to the eight standards in the National Safety and Quality Health Service (NSQHS) (Australian Commission on Safety and Quality in Health Care, 2015). However, these standards do not mention triaging, and so, the notion of triaging is not standardized. This is the reason why all dental practices that are accredited do not have the same triaging principles; some may lack triaging altogether. However, to ensure good care among patients in the community, effective triaging is a crucial skill for dental receptionists.

According to the Australian Dental Association website, although it is recommended that dental receptionists hold some form of qualification, dental receptionists in Australia are legally able to work without formal qualifications and are not governed by the Dental Board of Australia (Australian Dental Association, 2023). Obtaining these qualifications will require a combination of workplace assessment, as well as didactic learning to ensure that candidates are deemed vocationally competent. It is important to note that qualified dental assistants are able to perform reception duties, but not all dental receptionists are able to perform dental assisting duties. Therefore, it is important to identify areas of knowledge deficiency existing among dental receptionists. This may ultimately lead to incorporation of triaging into the education and qualifications of dental receptionists, resulting in better patient care. As there are no standardized guidelines for triaging dental patients in Australia, this research is important to determine whether individual clinics already have protocols in place. In addition, to date, there has been little research conducted on the efficacy of triaging patients with acute dental conditions by front desk staff at private, general dental practices.

To evaluate the care and services provided by health professionals, using patients as subjects in studies are especially useful. However, it is inconvenient to have real patients as subjects in a study as they are difficult to “standardize” for the duration of the study, and they may feel uncomfortable or unwilling to compromise their relationship with their clinician/health professional (Nestel et al., 2011). Simulated patients are individuals who are trained to act as a real patient and enact a standardized scenario. Another advantage of using the simulated patient method in a study is it minimizes the Hawthorne effect (Amaratunge et al., 2022; Björnsdottir et al., 2020). The Hawthorne effect is when an individual changes their behavior when they are aware they are being observed. Therefore, it would be ideal to undertake this quality assurance using covert simulated patient methodology. Before a simulated patient study can be implemented in the dental profession, it is important to understand knowledge of and perceptions about it from the profession itself. The aims of our study were to (1) explore dental staff knowledge of simulated patient methodology and support for its use for assessing triaging knowledge of dental receptionists and (2) to determine current triaging practices in dental practices.

## 2 METHODS

This cross‐sectional study consisted of an anonymous online questionnaire and is reported according to the Checklist for Reporting Results of Internet E‐Surveys (CHERRIES) (Eysenbach, 2004).

### 2.1 Inclusion criteria

All dentists and dental auxiliary staff currently employed in Western Australia were invited to participate through a survey link that was posted to dental specific social media groups. There are approximately 3000 registered dentists in Western Australia (general). There are approximately 1000 registered dental therapists, dental hygienists, dental prosthetists, and oral health therapists; with a 10% response rate, we would have 100 participants. However, it is important to note that dental assistants, practice managers, and dental receptionists are unregulated; as such, it is hard to accurately define the potential cohort size for these positions.

### 2.2 Survey design

The online questionnaire was anonymous, consisting of 19 questions on demographics [age, gender, profession, qualification, years of practice, occupational status], triaging, and knowledge about simulated patient methodology [knowledge, usefulness, context, ethics, debrief, and future context]. Questions about triaging and simulated patient methodology were developed by a team of experienced dentistry researchers and were based off guidelines from other countries such as the Scottish Dental Clinic Effectiveness Program (SDECP) guidelines from Scotland for triaging (Scottish Dental Clinical Effectiveness Programme, 2007). The questionnaire was provided in Supplement 1.

### 2.3 Survey administration

Surveys were delivered through Qualtrics^®XM^ software (Provo) using an anonymous online link and were available over 4 months (10/02/2021 to 21/06/2021). A reminder social media post was submitted on ADAWA website 3 months later as a follow‐up. As this questionnaire was conducted voluntarily, reminders were sent to everyone, regardless of whether they had completed the survey or not. The web link, along with the questionnaire information, was also provided through relevant social media (Facebook, LinkedIn, and Twitter). Participants could only access the questionnaire once they had consented to the study. Once they had completed the questionnaire, it was returned online directly to the researchers. No incomplete questionnaires were included in this study. For quality assurance, in addition to the “Prevent Ballot Box Stuffing” feature in Qualtrics®^XM^ software, IP addresses were manually checked to identify potential duplicate entries from the same user.

### 2.4 Statistical analysis

Normally distributed demographics were presented as both counts and percentages. All the responses were dichotomized except for the questions for which more than one answer could be submitted [types of triage questions]. To compare outcomes of the dichotomized variables across gender and year levels, Pearson Chi‐square tests were used. SPSS® version 27.0 (IBM Company) was used, and the statistical significance was set at *p* < .05. The percentage in the descriptive statistics was reported with the denominator for each question. To analyze the data, open‐ended questions in the free‐text response were imported to NVivo 12 (QSR International), a qualitative data management programme. Then, they were thematically analyzed by two researchers (V. L. and X. L.), and any discrepancies were resolved through discussion.

## 3 RESULTS

### 3.1 Demographics

The questionnaire was fully completed by 100 participants (Table 1). An additional 10 responses were partially completed and not included in the analysis. Responses were received by more females (71%), staff aged between 25 and 39 years (57%), dentists (46%), and predominantly private employees (60%).

**Table 1:** Percentage and counts outlining the demographics of all Western Australian dental staff who completed the survey (*n* = 100).

Demographics	Percentage	Count
*Age*		
18−24 years	7%	7
25−39 years	57%	57
40−60 years	32%	32
>60 years	3%	3
*Gender*		
Male	28%	28
Female	71%	71
Other, please specify	1%	1
*Occupation*		
Dental receptionist	6%	6
Dental assistant	21%	21
Practice manager	12%	12
Triage nurse	1%	1
Dental therapist, dental hygienist, oral health therapist	9%	9
Dentist	46%	46
Treatment coordinator, head nurse	6%	6
*Years in profession*		
<1 year	7%	7
1−5 years	29%	29
6−10 years	19%	19
11−20 years	21%	21
21−30 years	16%	16
>30 years	7%	7
*Qualifications*		
No qualifications	4%	4
Certificate III in dental assisting	1%	1
Certificate IV in dental assisting	21%	21
Diploma of leadership in health care practice	4%	4
Advanced diploma of leadership and management	1%	1
Certificate IV in business	1%	1
Bachelor of dentistry [BDS, BDSc]	19%	19
Master of dentistry [DMD, DDS]	25%	25
Others	22%	22
*Employee*		
Employee/private	60%	60
Employee/public	10%	10
Employer/private	7%	7
Employer/public	1%	1
Subcontractor	18%	18
Both private and public	3%	3

### 3.2 Triaging for dental appointments

The majority of participants who participated in the study currently triaged patients for dental appointments (82%) (Table 2). However, approximately three quarters of participants responded that they do not have dedicated triage staff in their dental clinic (74%). In terms of duration, two thirds of the dental staff spent about 1−5 min when triaging for appointments (66%). In terms of age, younger dental staff were more likely to state that there were dedicated triage personnel in their dental clinic (25−39 years = 64.7%; *p* = .02) and that they asked specific triage questions when they received a telephone call (25−39 years = 57.6%; *p* = .04). Furthermore, participants who reported having dedicated triage staff in their dental clinics took approximately 1−5 min when triaging their patients (*p* = .03). Years of working in the dental profession did not appear to make any difference when it came to triaging (*p* > .05).

**Table 2:** Dental staff and their response to triaging for dental appointments (*n* = 100).

Questions	Percentage	Count
*Do you (or the dedicated staff) in your dental clinic triage for appointments?*		
Yes	82%	82
No	13%	13
Unsure	4%	4
*Is there dedicated triage staff in your dental clinic?*		
Yes	25%	25
No	74%	74
Unsure	1.5%	1
*When triaging a patient over the telephone, how long is the conversation on average?*		
<1 min	16%	16
1−5 min	66%	66
5−10 min	15%	15
>10 min	3%	3

In terms of different questions asked (Table 3), of the 100 included participants, less than half enquired about what improves or worsens the symptoms (43%), medical history (29%), and past dental history (54%).

**Table 3:** Percentage and count of all responses to triaging questions (*n* = 100).

Question	Yes		No		May be	
	Percentage	Count	Percentage	Count	Percentage	Count
Who the appointment is for	100%	100	0%	0	0%	0
Purpose of the appointment (e.g., toothache, broken tooth, general dental check‐up)	97%	97	1%	1	1%	1
Site of problem (if any)	78%	78	6%	6	16%	16
Duration of problem (if any)	57%	57	25%	25	18%	18
What resolves or worsens the problem (if any)	43%	43	32%	32	25%	25
COVID‐19 questionnaire	60%	60	26%	26	13%	13
Patient details	94%	94	3%	3	3%	3
Medical history	29%	29	53%	53	18%	18
Past dental history	19%	19	60%	60	21%	21
Private health status	54%	54	37%	37	9%	9

### 3.3 Perception and attitude toward simulated patient studies

Data are presented in Table 4. Most participants had not heard of simulated patient studies (74%) and approximately two‐thirds said they would find it useful (65%). In terms of undertaking simulated patient studies, about 27% of the participants believed that assessing for acute dental triaging may be useful. Regarding the overall approach, 40% of staff were neutral about the idea and 42% of staff were either moderately or extremely supportive. Significantly more male staff were concerned about issues relating to covert simulated patient studies (*p* = .0328), compared to females. When asked about ways to debrief following a simulated patient visit, most participants indicated they would prefer this to occur immediately after the visit was conducted (68%). It was also found that female participants preferred some form of debriefing after simulated patient studies had been conducted (*p* = .04). In terms of professional sectors, private employees were more likely to ask a greater number of triage questions compared to public employees (*p* < .00001). In addition, private employees indicated preference for some form of debriefing if they had been involved as a participant in simulated patient studies (*p* = .000533).

**Table 4:** Percentage and count of participant perception and attitudes toward studies using simulated patient methodology (*n* = 100).

Questions	Percentage	Count
*Have you heard about simulated patient studies?*		
Yes	24%	24
No	74%	74
May be	1%	1
I do not know	1%	1
*Do you believe that simulated patient studies may be useful?*		
Definitely not	0%	0
Probably not	4%	4
Might or might not	31%	31
Probably yes	47%	47
Definitely yes	18%	18
*What type of simulated patient study do you think may be useful in dental clinical settings?*		
Treatment options provided by the dentist	19%	19
Acute dental triaging by the front desk staff	27%	27
Appointment scheduling by the front desk staff	21%	21
Handling of complaints by the dental clinic staff	18%	18
Cleanliness/hygiene status of the dental clinic	13%	13
Others [attitudes of staff, phone shoppers]	2%	2
*Opinion of covert approach*		
Extremely concerned	4%	4
Moderately concerned	13%	13
Neutral	40%	40
Moderately supportive	29%	29
Extremely supportive	13%	13
*Reasoning for support/against covert approach*		
Lack of informed consent	17%	17
Improvement of profession as a whole	32%	32
Ethical concerns otherwise not stated	18%	18
Time consumption from work	13%	13
Anonymity issues	17%	17
Other [Stress, improved customer service]	2%	2
*Ways to debrief simulated patient studies*		
Explain after the scenario has been completed	68%	68
No debriefing needed	7%	7
Debrief in a professional setting newsletter (e.g. Australian Dental Association Bulletin)	16%	16
Others	9%	9

Thematic analysis of open‐ended questions about simulated patient methodology revealed four themes: debriefing, support, education, and realism.

### 3.4 Debriefing

A few participants suggested debriefing once the study had been completed.

“Come out with scenario base and collect the front desk answer and acknowledge at the end.” [Dental assistant 17]

“Call the clinic, once complete, call the owner/manager to see if they would like to discuss findings.” [Dental receptionist 3]

### 3.5 Support

Participants provided suggestions on ways covert simulated patient studies could be conducted that they would find acceptable. This is to show a degree of transparency.

“Announce that this will be occurring with Australian Dental Association Western Australia email to members” [Practice manager 4]

*“The practice manager or owner can be aware that this will occur in their clinic, however not disclose to other staff.” [Dental assistant 10]*

“As long as they know they will be receiving these calls but aren't told who the callers are going to be or what they sound like, the study can be conducted ethically.” [Dentist 7]

Some participants showed interest and slight curiosity by showing support.

“Call up with a toothache and see how they triage you.” [Dental receptionist 1]

A few participants showed support for covert simulated patient studies, having no problems with taking a covert approach.

“I don't really see the problem. I can understand how it can be for some.” [Oral Health Therapist 2]

However, there was one participant who had trouble determining whether a covert approach was an issue.

“I am at a fence. Not sure why? I know it is important but I can't put my head around it. Hard to come up with an answer.” [Dentist 3]

### 3.6 Education

Some participants viewed simulated patient studies as a form of quality assurance.

“…To use as data collection for improved staff training…” [Practice manager 1]

Some participants saw the value of this type of study to provide authentic data to improve practice by dissemination of results and learnings.

“Australian Dental Association auditing safety and quality COVID triage minimise cancellations” [Dentist 30]

“Australian Dental Association webinar” [Dentist 10]

### 3.7 Realism

Some participants suggested the importance of using real‐life scenarios.

“…keep scenario as real as possible…” [Treatment coordinator 2]

## 4 DISCUSSION

In this study, we investigated the knowledge and attitudes of dental staff toward simulated patient studies in Western Australia. We found that while most participants were unfamiliar with simulated patient methodology, the majority thought these studies would be useful. The results of our study are consistent with previous studies that have been conducted (Rhodes & Miller, 2012; Steinman, 2014). One such example is a study by Rhodes, where it was found that simulated patient studies can be ethically justified when it is used as an educational tool to better the training and education of the profession given that participants are deidentified and confidentiality is maintained (Rhodes & Miller, 2012). Younger participants, private employees, and those who asked triage questions for appointments were more likely to be aware of the existence of simulated patient studies. This study also found that employees of private dental practices asked more specific triage questions compared to employees working in the public sector. Employees of private dental practices also indicated they would prefer to have some form of debriefing (immediate or delayed) as they felt it was an educational experience that helped better their performance, which is consistent with the literature (Xu et al., 2012).

These results indicate the possibility of integrating simulated patient studies into the training and credentials of dental receptionists, which is consistent with studies performed in the pharmacy field (Schneider et al., 2009, 2010). An example of a simulated patient study that has been used to improve care is a study assessing the provision and supply of salbutamol by Schneider and colleagues (Schneider et al., 2009, 2010). This study discovered that 47% of salbutamol sales occurred without input by a registered pharmacist. This discovery led to changes in the legislation to specify that salbutamol sales needed to be “direct personal sale” by a registered pharmacist. In addition to that, intern pharmacists were given the responsibility of developing and implementing an intervention as part of their intern training program. This indicates that simulated patient studies can have a significant impact on initiating changes to policies and guidelines, ultimately resulting in enhanced patient care (Schneider et al., 2010). However, further research is needed to evaluate the efficacy of triaging patients with acute dental conditions by reception staff at private or public, general dental clinics.

Evaluating the care and services offered by health care professionals is particularly beneficial by employing patients as subjects in research. Nonetheless, utilizing actual patients as participants in a study presents challenges due to the complexity of standardizing their involvement throughout the research period and their potential discomfort or reluctance to jeopardize their rapport with health care providers. On the other hand, simulated patients are individuals trained to replicate patient behavior and perform within a predetermined scenario. As previously discussed, employing simulated patient methodology offers the advantage of reducing the Hawthorne effect (Nestel et al., 2011). There have been many simulated patient studies conducted in the pharmacy setting (Schneider et al., 2009, 2010, 2013). The quality reporting checklist that is used by researchers when reporting simulated patient studies is the Checklist for Reporting research using Simulated Patient (CRiSP) methodology (Amaratunge et al., 2021). More recently, CRiSP checklist has been refined as CRiSPHe (checklist for reporting research using a simulated patient methodology in Health) to be more universal for other health professionals (Park et al., 2024). The transparency and thorough reporting of simulated patient studies in the pharmacy field allow identification of the transferability of the checklist to other disciplines, such as when assessing the triaging ability of dental receptionists. Simulated patient studies have been shown to provide the initiative and evidence to bring about changes in regulations, guidelines, and legislation (Schneider et al., 2010).

### 4.1 Implications to future practice

Dental reception staff are the first point of contact for patients with acute dental issues; therefore, it is crucial that they are able to effectively triage patients to enable timely access to care, mitigating progression to serious consequences (McGuckin, 2020). Such consequences include systemic infection, pulpal involvement, fistulas, and abscesses (Abbott & Yu, 2007). Currently, there is a lack of simulated patient studies assessing the triage abilities of dental receptionists within private, general dental clinics. Recognizing the areas of knowledge deficiency among dental receptionists could potentially result in integration of triage training into the education for receptionists. In a previous retrospective study which evaluated the triaging effectiveness of dental nurses, the majority of patients were effectively managed by a phone call with the triage nurse who had the knowledge and skills to correctly identify the patient's symptoms and condition (Cowell et al., 2020). The study showed that triaging patients is effective when the individual triaging has sufficient knowledge to identify and manage patients with acute dental conditions so that they can effectively access the care they need. However, the effectiveness of triaging needs to be evaluated. One way this could be achieved is by conducting a study with simulated patient methodology to assess data on triaging knowledge and skills of dental receptionists.

### 4.2 Strengths and limitations

Due to the unexpected nature of the COVID‐19 pandemic, our study has been limited to a retrospective format, which means that a higher probability of confounding factors and bias has been included. Moreover, our methodology has been limited to recruiting 100 respondents, limiting the generalizability and reliability of our findings. Therefore, it was challenging to generalize the dental clinic workforce in Western Australia accurately. However, these results are useful as a pilot study and should be explored in larger sampled research.

The information about the survey was disseminated through a link placed on social media groups, particularly for dentists and dental auxiliary staff currently working in Western Australia. Recruiting participants based on social media could not provide a complete, adequately representative picture of the entire dental workforce. This is due to recruiting only on social media, resulting in respondents likely being biased toward the ones most active on this platform and failing to cover other, less active groups of users professionally or not using it as a tool of their daily activities.

### 4.3 Future research

Given the limitations identified in the current study, it is possible to develop the research further to explore whether the theoretical framework should be specified and whether the argumentation concerning the role of unregulated professionals in dental practices through performing triaging, such as dental receptionists, should be detailed. In particular, one may develop detailed research and explore who is the better professional to perform triaging in dental practices, whether it is the responsibility of the dental receptionist or specially qualified dental assistant, explore their difference in initial trial assessment, and outline the benefits of both staffing models for patient care and productivity.

In addition, no triaging protocol is available throughout Australia; other researchers can build upon the study results to conduct more research exploring successful triaging practices and training. In particular, this research may involve developing and exploring several triage protocols' impact on patient outcomes and patient satisfaction with the provided care. Furthermore, it may help determine the feasibility and benefits of using simulated patient studies in dental practices to train dental staff in triaging, enhancing the implementation process of research‐based policy and practice guidelines in the dental field.

## 5 CONCLUSION

In conclusion, the results of the exploratory study, which consists of a small sample of dental receptionists working at private general practices, emphasize the apparent value of simulated patient studies for expanding the training and competencies of dental receptionists to promote better patient care. While the sample size of our pilot study was small, limiting the generalization of our findings, the present results can be viewed as an essential preliminary assessment that establishes the need for further research in this area. More knowledge and awareness about the use and importance of simulated patient methodologies need to be gained, as illustrated by the survey conducted in Western Australia. However, we observe a positive attitude toward using simulated patients, which indicates the readiness of the sector to adopt such methodologies. Future research should include expanding the participant pool to increase its diversity and size. This would help to obtain a more comprehensive understanding of the effectiveness of different triaging protocols and their impact on patient outcomes and satisfaction. In addition, future studies could focus on the logistical considerations of integrating simulated patient studies into regular dental practice to evaluate its feasibility and the effect on long‐term mechanisms in real‐world applications. This research path can contribute to formulating robust, empirically informed triage guidelines and, consequently, enhance the quality of care at the first point of patient contact at dental clinics.

## AUTHOR CONTRIBUTIONS

**Viduni Liyange**: Conception and design, analysis and interpretation, revising the work, final approval. **Xin Rong Low**: Conception and design, analysis and interpretation, drafting the work, revising the work, final approval. **Joon Soo Park**: Conception and design, acquisition of data, drafting the work, revising the work, final approval. **Hien C. Ngo**: Rrevising the work, final approval. **Rhonda Clifford**: Revising the work, final approval. **Liza Seubert**: Conception and design, drafting the work, revising the work, final approval.

## CONFLICT OF INTEREST STATEMENT

The authors declare no conflict of interest.

## ETHICS STATEMENT

Ethics approval to conduct this study was obtained from the Human Research Ethics Committee at the University of Western Australia (Approval Number—2022/ET000301).

## Supporting information

Supporting information.

## Data Availability

Data available on request from the authors.
