# Supplementary material for: Exploring the usability of simulated patient methodology in dental clinics in Western Australia: A pilot survey
Source: Clin Exp Dent Res. 2024 Jul 5;10(4):e906. doi: 10.1002/cre2.906 (PMC11226537; doi:10.1002/cre2.906)
Supplement: Supplementary file 1 — Supporting information. [file CRE2-10-e906-s001.pdf]

Supplement 1 - Questionnaire used for the pilot survey

Questionnaire

Demographics

Age

16-18 years
18-24 years
25-39 years
40-60 years
>60 years

Gender

Male
Female
Non-binary / third gender
Other, please specify

Profession

Dental Receptionist
Dental Assistant
Practice Manager
Triage Nurse
Dental therapist, Dental hygienist, Oral health therapist
Dentist
Other, please specify

Years of practice in profession?

< 1 years
1 - 5 years
6 - 10 years
11 - 20 years
21 - 30 years
> 30 years

What is your highest level of qualification?

No qualification
Certificate III in Dental Assisting

Certificate IV in Dental Assisting

Diploma of Leadership in Healthcare Practice

Advanced Diploma of Leadership and Management

Certificate IV in Leadership & Management

Certificate IV in Business

Certificate IV in Medical Practice Assisting

Certificate III in Business Administration (Medical)

Bachelors dental [BDS, BDSc]

Masters dental [DMD, DDS]

Masters of Business Administration [MBA]

Others, please specify

What describes your professional status?

Employee / Private

Employee / Public

Employer / Private

Employer / Public

Sub-contractor

Others, please specify

Do you (or the dedicated staff) in your dental clinic triage for appointments?

Yes

No

Unsure

Is there a dedicated triage staff in your dental clinic?

Yes

No

Unsure

When triaging a patient over the telephone, how long is the conversation?

< 1 minute

1-5 minutes

5-10 minutes

> 10 minutes

When making appointments, do you or the staff ask the following? **[Please select all the options provided]**

	Yes	No	Maybe
Who the appointment is for	○	○	○
Purpose of the appointment (e.g. toothache, broken	○	○	○

	Yes	No	Maybe
tooth, general dental check-up)			
Site of problem (if any)	○	○	○
Duration of problem (if any)	○	○	○
What resolves or worsen the problem (if any)	○	○	○
COVID-19 questionnaire	○	○	○
Patient details	○	○	○
Medical history	○	○	○
Past dental history	○	○	○
Private health status	○	○	○

Simulated patient study "Mystery shopper" involves a simulated patient [an individual who is trained to enact a predetermined scenario in such a way that they are indistinguishable from genuine patients].

Before today, have you heard about simulated patient study?

Yes
No
Maybe
I do not know

Do you believe that simulated patient study may be useful?

Definitely not
Probably not
Might or might not
Probably yes
Definitely yes

What type of simulated patient study would you think may be useful in the dental clinical settings? **[You can choose more than one answer]**

Treatment options provided by the dentist
Acute dental triaging by the front desk staff
Appointment scheduling done by the front desk staff
Handling of complaints by the dental clinic staff
Cleanliness/Hygienic status of the dental clinic
Other, please specify

Simulated patients studies are useful in that they are a covert [not openly acknowledged] approach to assess professional practice without the Hawthorne effect (whereby an individual changes their behaviour due to being observed)

What are your thoughts on covert approach?

Extremely concerned

Moderately concerned

Neutral

Moderately supportive

Extremely supportive

If you are concerned or supportive about the covert approach, please provide your reasoning **[You can choose more than one answer]**

Lack of informed consent

Improvement of profession as a whole

Ethical concerns otherwise not stated

Time consumption from their work

Anonymity issues

Other, please specify

How do you think we can debrief if we were to perform simulated patient study in your dental clinic?

Explain after the scenario has been completed

No debriefing needed

Debrief in a professional setting newsletter (e.g. Australian Dental Association Bulletin)

Other, please specify

In the upcoming future we are planning to undertake a simulated patient study in a dental clinic where we ring to assess the knowledge of the front desk staff at the private general dental clinic in triaging acute dental conditions.

How do you think we can approach this ethically? [Please explain]
